# Conserved Motifs and Prediction of Regulatory Modules in *Caenorhabditis elegans*

**DOI:** 10.1534/g3.111.001081

**Published:** 2012-04-01

**Authors:** Guoyan Zhao, Nnamdi Ihuegbu, Mo Lee, Larry Schriefer, Ting Wang, Gary D. Stormo

**Affiliations:** * Department of Genetics, Washington University School of Medicine, St. Louis, Missouri 63110; † Brigham Young University, Provo, Utah 84602

**Keywords:** *cis*-regulatory element, *cis*-regulatory module, transcription factor, transcriptional regulation, *Caenorhabditis elegans*

## Abstract

Transcriptional regulation, a primary mechanism for controlling the development of multicellular organisms, is carried out by transcription factors (TFs) that recognize and bind to their cognate binding sites. In *Caenorhabditis elegans*, our knowledge of which genes are regulated by which TFs, through binding to specific sites, is still very limited. To expand our knowledge about the *C. elegans* regulatory network, we performed a comprehensive analysis of the *C. elegans*, *Caenorhabditis briggsae*, and *Caenorhabditis remanei* genomes to identify regulatory elements that are conserved in all genomes. Our analysis identified 4959 elements that are significantly conserved across the genomes and that each occur multiple times within each genome, both hallmarks of functional regulatory sites. Our motifs show significant matches to known core promoter elements, TF binding sites, splice sites, and poly-A signals as well as many putative regulatory sites. Many of the motifs are significantly correlated with various types of experimental data, including gene expression patterns, tissue-specific expression patterns, and binding site location analysis as well as enrichment in specific functional classes of genes. Many can also be significantly associated with specific TFs. Combinations of motif occurrences allow us to predict the location of *cis*-regulatory modules and we show that many of them significantly overlap experimentally determined enhancers. We provide access to the predicted binding sites, their associated motifs, and the predicted *cis*-regulatory modules across the whole genome through a web-accessible database and as tracks for genome browsers.

The development of an organism is largely controlled by transcriptional regulation that determines where and when every gene is expressed. A first step toward the understanding of how genomic DNA controls the development of an organism is to understand the mechanisms that control differential gene expression. Transcriptional regulation is carried out by transcription factors (TFs) via their binding to specific DNA sequences. Binding sites of TFs can be represented as consensus sequences, but position weight matrices (PWMs) provide a more quantitative description of the specificity of a TF (Stormo 2000). Currently our knowledge of the TFs and their binding sites is very limited. For example, the human genome has greater than 2000 predicted TFs (Lander *et al.* 2001), but only a few hundred have quantitative models of their specificity, primarily determined by computational tools that have been developed to facilitate the identification of PWMs for TFs (reviewed in GuhaThakurta 2006). Furthermore, although computational methods can successful identify binding sites that are bound by a particular TF *in vitro*, most of the predicted binding sites are not functional *in vivo* (Li *et al.* 2011; Whittle *et al.* 2009). In previous studies, authors have shown that TF binding sites tend to cluster together to direct tissue/temporal-specific gene expression (Arnone and Davidson 1997; Kirchhamer *et al.* 1996). These clusters of binding sites that regulate expression are referred to as *cis*-regulatory modules (CRMs). Clustering of TF binding sites, along with phylogenetic conservation and other measures of “regulatory potential,” have been widely used in the computational prediction of CRMs and is a more reliable indicator of *in vivo* regulatory function of DNA sequences (Blanchette *et al.* 2006; Ferretti *et al.* 2007; King *et al.* 2005; Kolbe *et al.* 2004; Sinha *et al.* 2006; Taylor *et al.* 2006; Wasserman and Sandelin 2004).

*Caenorhabditis elegans* has been an important model organism for studying development and was the first metazoan with a completely sequenced genome (C. elegans Sequencing Consoortium 1998). Although a few promoter regions have been studied in detail (Ao *et al.* 2004; Gaudet and Mango 2002; Krause *et al.* 1994; McGhee *et al.* 2007, 2009), most transcriptional regulatory interactions remain unknown. Recently projects have been undertaken to gain a more comprehensive view of which TFs regulate which promoters using experimental approaches to identify their interactions directly (Celniker *et al.* 2009; Deplancke *et al.* 2006; Gerstein *et al.* 2011), but those are still in early phases. A complementary approach is to identify noncoding segments of the genome that are conserved across species and are likely to contain regulatory elements (reviewed in Wasserman and Sandelin 2004).

There are several previous works on regulatory motif prediction in *C. elegans* that focused on sets of genes that are expressed under specific conditions or in specific tissues (Ao *et al.* 2004; Gaudet *et al.* 2004; GuhaThakurta *et al.* 2002, 2004). A recent report compared eight nematode species and identified regions from more than 3800 genes that are conserved between *C. elegans* and at least three other species; those are cataloged in their cisRED database (Sleumer *et al.* 2009). In this article we performed a genome-wide *cis*-regulatory element identification using PhyloNet (Wang and Stormo 2005), which systematically identifies phylogenetically conserved motifs that also occur multiple times throughout the genome and are likely to define a network of regulatory sites for a given organism. The first step of this approach is similar to that used for cisRED, *i.e.*, identifying segments conserved across multiple species, but then it further compares all such conserved regions to each other to identify those associated with multiple genes.

Applying PhyloNet on 2-kb intergenic regions from the genomes of *C. elegans*, *C. briggsae*, and *C. remanei* leads to the identification of *cis*-regulatory elements from various functional categories. We identified core promoter elements, TF binding sites, splicing sites, poly-A signals, and others. In addition, for each regulatory element, PhyloNet identified a set of genes that are potentially regulated by the motif. Gene functional enrichment and expression coherence analysis under several conditions provide strong support that most of the motifs are functional elements that are responsible for the regulation of the target genes. The instances of these predicted *cis*-regulatory elements within the promoter region sequences are highly clustered. Based on this observation we developed a program, CERMOD, to predict new CRMs. Comparison between the predicted modules with experimentally characterized modules shows high sensitivity with 83.2% (129/155) of experimentally characterized modules. For genes with experimentally determined CRMs, 23.4% (219/934) of our predicted modules are located within experimentally defined regions. This is a lower bound of predictive accuracy because most of our predicted modules could be real but are located within promoter regions that have not been tested.

## Material and Methods

### Genome sequences

The chromosomal sequence and the gene structures of *C. elegans* (C. elegans Sequencing Consortium 1998) (WS170) and *C. briggsae* (Stein *et al.* 2003) genome are downloaded from the Wormbase ftp-site (ftp://ftp.wormbase.org/pub/wormbase/genomes/). Upstream, intergenic region sequences of up to 2 kb in length were obtained. (If the distance to the upsteam gene is less than 2 kb, only the intergenic region was obtained. We refer to the sequences as “2-kb upstream regions” throughout the article.) *C. remanei* sequence and annotation were produced by the Genome Sequencing Center at Washington University School of Medicine in St. Louis and were obtained from http://genome.wustl.edu/pub/organism/Invertebrates/Caenorhabditis_remanei/.

### Identification of orthologs of *C. elegans* genes

*C. briggsae* orthologs of *C. elegans* genes were obtained from WormBase (ftp://ftp.wormbase.org/pub/wormbase/datasets-published/stein_2003/orthologs_and_orphans/orthologs.txt.gz). To identify *C. elegans* orthologous genes in the *C. remanei* genome, we used the NCBI BLAST program (version 2.0) (Altschul *et al.* 1990) to compare all annotated protein coding gene sequences in the *C. remanei* genome with that in the *C. elegans* genome. Two genes are defined to be orthologous if all of the following three conditions are met: (i) their protein sequences are reciprocal best BLASTP hits between two genomes; (ii) the BLASTP E-value is lower than 1E-10; and (iii) the BLAST alignment covers ≥60% of the length of at least one sequence. The promoter region sequences of all genes in the orthologous gene set that contain both *C. briggsae* and *C. remanei* orthologs of *C. elegans* gene were retrieved. The promoter region is defined as intergenic sequences upstream of translational start site ATG from −1 to sequence up to the next coding gene, but no more than 2 kb. Each sequence group of orthologous genes forms a data entry. For *C. elegans* genes that are in operons (Blumenthal *et al.* 2002), we only considered the first genes in the operons.

### Motif identification and consolidation

We used PhyloNet, a program that systematically identifies phylogenetically conserved motifs and defines a network of regulatory sites for a given organism to search for conserved regulatory elements (Wang and Stormo 2005). PhyloNet was run with options s = 1, iq = 20, id = 20, and pf = 10. Up to 10 predicted *cis*-regulatory elements are reported for each intergenic region. *Cis*-regulatory elements are represented by PWMs (Stormo 2000), and each matrix is associated with a set of genes that are potentially regulated by this element (gene cluster).

The initial motifs generated by PhyloNet are redundant because each gene is used as a query and different gene queries can generate very similar motif profiles and target gene clusters. To remove redundancy of the whole genome motif profile set, we used the average log likelihood ratio (ALLR) statistic (Wang and Stormo 2003) to determine the similarity between motif profiles. ALLR statistics are implemented in MatAlign-v4a (Wang and Stormo; http://stormo.wustl.edu/MatAlign/). Similarity of two motif profiles is determined by the ALLR scores of each pair of motif profiles and the length of the aligned part of the two motifs. To determine the best parameters for clustering PWMs, we analyzed matrices in the TRANSFAC database (Matys *et al.* 2003). TRANSFAC version 10.2 contains 811 PWMs, 540 of which have known binding factors that are classified at the family level. PWM similarity is measured with two parameters: ALLR score and OLAP score, which is the percentage of the two PWMs that overlap. At each ALLR and OLAP score cutoff value, we compare each of the 540 matrices with all of the others to determine the score distributions. From this information, we calculate sensitivity and specificity for classifying each PWM into the correct family at each ALLR and OLAP cutoff value. Our results suggest that ALLR > 6.57, OLAP > 68.1% gives the best specificity. For all PhyloNet output matrices, the best one is picked first (the one with the highest total ALLR score in the PhyloNet output). It is compared with the rest of the matrices using ALLR statistics, and any matrix that appears redundant to the chosen matrix is removed. Then, the second best one is picked, and the process is repeated until all the matrices have been analyzed.

### Calculation of functional enrichment of target genes sharing the same motif

We tested the functional enrichment of target genes of each motif profile based on Gene Ontology (GO). GO terms and annotations of *C. elegans*, were downloaded from WormBase (GO terms was downloaded on October 25, 2006. GO.WS170.txt was downloaded on April 30, 2007). All genes sharing the same GO term are clustered. Based on GO term hierarchies, we added all genes in the children GO terms to the current GO term gene cluster. The cumulative hypergeometric distribution (Tan *et al.* 2005; Tavazoie *et al*. 1999) is used to calculate the *P*-value of observing the number of genes associated to a motif profile and enriched in a particular GO term.

### Calculation of microarray expression profile coherence

Microarray expression profiles are downloaded from the Gene Expression Omnibus. Expression coherence score and threshold distance were calculated as described (Pilpel *et al.* 2001). We define the gene clusters to have significant expression coherence when their *P*-value < 0.05 after correction for multiple tests.

### CRM identification

To identify DNA regions enriched for predicted motifs, we first identify all predicted sites for all the motifs using Patser (Hertz and Stormo 1999) using default cutoff scores. Then, we calculate the average number of binding sites per position in the sequence and Z score for each position. We identify those peak positions that have a Z score ≥ 3.09 (corresponding to *P*-value = 0.001). For each peak position, we extend it in both 5′ and 3′ direction if the next Z score > 0 position is less than 30 bp away (the longest motif length). Peak positions used in a previous extension step are not extended.

## Results and Discussion

This section is divided into four subsections:

1. Overview of the conserved motifs identified by PhyloNet.
2. Correspondence between the motifs and several different types of experimental data to assess their likely functions.
3. Using the motifs to predict CRMs across the entire intergenic regions of *C. elegans* and an assessment of the accuracy of those predictions.
4. A description of the database of exemplar sites and motifs and of the genome browser that facilitate access to the sites, motifs and module predictions.

### Overview of the conserved motifs identified by PhyloNet

To systematically identify conserved elements in *C. elegans*, we used the genome sequences from *C. briggsae* and *C. remanei*. We obtained 11,860 *C. briggsae* orthologs and 12,466 *C. remanei* orthologs for 16,544 *C. elegans* genes. Some *C. elegans* genes are organized into operons and genes in operons share a common promoter sequence that allows coordinated expression of the genes. After removing the distal genes in operons, as annotated in Wormbase (http://www.wormbase.org/), 10,491 and 11,064 of the *C. elegans* genes have *C. briggsae* and *C. remanei* orthologs, respectively. A total of 9356 genes that have both *C. briggsae* and *C. remanei* orthologs were used for further analysis.

Current evidence indicates that *C. elegans* regulatory regions are fairly compact and most known regulatory elements occur within 2 kb upstream of the coding region of the gene (Dupuy *et al.* 2004; Sleumer *et al.* 2009; Zhao *et al.* 2007). We retrieved up to 2 kb upstream promoter region sequences for all of the genes with orthologs in both species. Each *C. elegans* gene and its orthologs form a data entry that contains three promoter regions. For each data entry, PhyloNet (Wang and Stormo 2005) was applied to query the database and up to 10 most significant predicted motifs, represented as PWMs (Stormo 2000), were obtained for further analysis. Because of the greedy and reciprocal nature of the PhyloNet algorithm, where each promoter region sequence serves as the query for a BLAST-like alignment to every other promoter region sequence, these initial predicted motifs in the PhyloNet output files are highly redundant. We took two steps to consolidate predicted motifs. The first step compares matrices in each query output file to consolidate matrices that significantly overlap. This step results in a total of 36,953 PWMs, an average of 3.95 PWMs for each *C. elegans* promoter region sequence. This set of sites is called the exemplar sites, those identified by PhyloNet as being conserved in the three species and significantly similar across multiple genes. From the initial set of nearly 20 Mbp in candidate regions from C. elegans, the exemplar sites cover a total of 3,695,282 bp, which is approximately 18% of the intergenic regions considered.

The second step is to consolidate PWMs based on motif similarity to generate the final set. This step is challenging because our goal is to find *cis*-acting regulatory motifs that correspond to all of the *trans*-acting regulatory factors, but there is not a simple one-to-one relationship between them. One complication is that TFs from the same structural family often bind to highly similar DNA target sequences (Luscombe *et al.* 2000), and it can be difficult to separate sites for different TFs based on the conserved motifs alone. Several computational approaches have been developed to quantify similarities between PWMs (Kielbasa *et al.* 2005; Schones *et al.* 2005; Wang and Stormo 2003), and to use this information to classify the structural class of mediating TFs for novel motifs (Kielbasa *et al.* 2005; Narlikar and Hartemink 2006; Sandelin and Wasserman 2004; Schones *et al.* 2005). We use the ALLR (Wang and Stormo 2003) to cluster motifs into distinct sets. Although Mahony *et al.* (Mahony *et al.* 2007) did not find ALLR to be the best statistic for assigning motifs to TF structural classes, our most challenging goal is to distinguish similar motifs from the same class, for which ALLR is well suited.

Our stringent criteria (see *Materials and Methods*) allow only very similar motifs being clustered together. This gives us confidence that we have not merged motifs for different TFs but has the disadvantage that we may have several distinct PWMs remaining for the same TF. This is certainly the case as the second consolidation step leaves us with 4959 distinct motifs with lengths between 5 and 30 bases, many more than the proposed number of approximately 940 *C. elegans* TF genes (Reece-Hoyes *et al.* 2005). These motifs cover 3,442,144 bp and have an average length of about 15 bp. We find other types of known motifs besides TF binding sites (see next section), but in addition the motifs probably contain sites for combinations of TFs that we have not separated into distinct subsites. These consolidated PWMs are all very significant (*P* < 10^−10^), and each is associated with a set of genes that are potentially regulated by this motif. Each consolidated PWM is associated with a set of exemplar sites and a gene list. The gene lists range from 3 to 7724 genes. We expect the exemplar sites for each PWM to be an incomplete set of binding sites for the associated factor because less than half of the *C. elegans* genes are used in our initial promoter region sequence set and because, even for orthologous genes, some sites will not be conserved across the different species. We can use the PWMs to predict other potential binding sites for the associated factor. These predicted sites should provide a more comprehensive list of binding sites, and regulated genes, for each PWM, but will likely also include some false predictions.

### Correspondence between the motifs and several different types of experimental data

**Figure 1:**
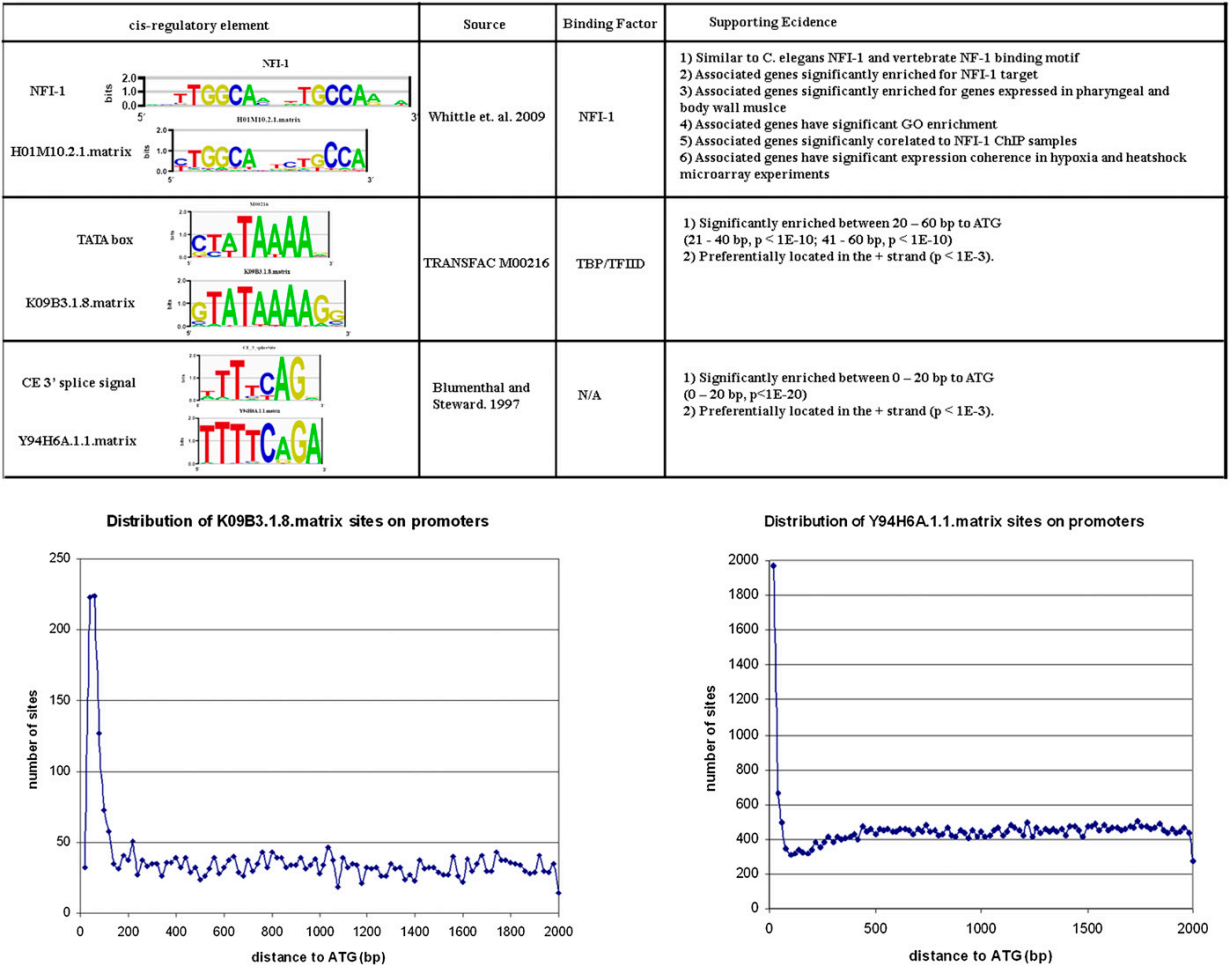
Examples of three different classes of conserved elements and supporting evidence. Top: Sequence logo, reference, binding factor, as well as supporting evidence for NFI-1, TATA box, and *C. elegans* 3′ splice/*trans*-splicing signal. Bottom: Distribution of K09B3.1.8.matrix and Y94H6A.1.1.matrix sites on promoters.

Intergenic regions contain different kinds of regulatory elements. We are particularly interested in TF binding sites involved in controlling gene expression, but other elements are also obtained in our set of conserved motifs. Figure 1 shows three different classes of conserved elements that emerge from this analysis. The PWM H01M10.2.1 is likely to represent the binding motif for the TF nuclear factor I-1 (NFI-1) on the basis of several types of evidence: (1) it is highly similar to the documented NFI-1 binding site (Whittle *et al.* 2009) and the vertebrate NF-1 binding site (TRANSFAC AC number: M00056); (2) the gene cluster associated with the H01M10.2.1 matrix is significantly enriched for the known NFI-1 target genes (Whittle *et al.* 2009) (*P* < 10^−14^); (3) the gene cluster associated with the H01M10.2.1 matrix is significantly enriched for genes that are expressed in pharynx (*P* < 3 × 10^−5^) and body wall muscle (*P* < 7 × 10^−3^), which is consistent with observed NFI-1 expression in C. *elegans* (Lazakovitch *et al.* 2005, 2008); (4) H01M10.2.1 is significantly correlated to NFI-1 ChIP samples (*P* < 10^−6^; *t*-value ~15.6); and (5) the gene cluster associated with the H01M10.2.1 matrix is significantly enriched for GO terms that are consistent with NFI-1’s function.

A second type of element we obtain is a core promoter motif such as the TATA-box (Figure 1). K09B3.1.8 matrix is very similar to the TRANSFAC TATA box PWM (M00216) and, unlike most transcript factors binding sites, it is significantly biased in its location and its orientation. It is significantly overrepresented near the translational start site ATG, in positions between 21-40 (*P* < 10^−10^) and 41-60 (*P* < 10^−10^) nucleotides upstream of the ATG. It is also preferentially located on the + strand (*P* < 10^−3^), as expected for a core promoter element. A third type of element we find are those related to RNA processing. In *C. elegans* more than half of pre-mRNAs are subject to SL1 *trans*-splicing (Blumenthal and Steward 1997). The *trans*-splice site consensus on the pre-mRNAs is the same as the intron 3′ splice site consensus. Y94H6A.1.1 matrix is significantly similar to the *C. elegans* *trans*-splice/3′ splice signal. It is significantly overrepresented near the translational start site ATG (Figure 1). However, different from the TATA box, it is preferentially located between 0 and 20 nucleotides upstream of ATG (*P* < 10^−20^). Trans-splicing occurs close to the start codon in *C. elegans*, with 49% of transcripts analyzed containing a spliced leader sequence within 10 nucleotides of the initiator AUG (Lall *et al.* 2004). In addition, Y94H6A.1.1.matrix is preferentially located on the + strand (*P* < 10^−3^) as expected for a splicing signal. The fact that the AG at the end of the motif is not completely conserved indicates that some of the exemplar sites will not be true sites and indicates one of the limitations of completely automated methods. We did not find a motif that represents the 5′ splice signal, which is consistent with the presence of 3′ but not 5′ splice signal in front of ATG in the case of *trans*-splicing.

Besides identifying PhyloNet PWMs that correspond to motifs for known factors as described above, we can assess whether the genes associated with any PWM are significantly correlated with specific biological assays. In the following sections we consider data from four different approaches: (1) TF binding data, such as ChIP-chip and ChIP-seq experiments that identify binding locations for specific TFs; (2) expression data, such as microarrays that measure gene expression patterns under specific conditions or specific genetic backgrounds; (3) tissue-specific expression patterns of genes using GFP-fusions; and (4) enrichment for specific classes of genes using GO classifications for genes. Significant correlations between the genes selected by any of those methods and genes associated with one of our PWMs provide supporting evidence that the PWM represents a regulatory motif.

#### Location analysis:

Regions in the genome in which TFs bind *in vivo* can be determined experimentally by expressing tagged *C. elegans* TFs that then are cross-linked to chromatin and their locations determined by either array hybridizations (ChIP-chip) or sequencing (ChIP-seq). We compare those experimentally determined binding locations to the predicted occupancy for each PWM on each promoter region sequence. The predicted occupancy is calculated by scoring each position in the promoter sequence with the PWM and summing the exponentiated scores:

$$Occ\left(P_{j},M_{k}\right)\propto \sum_{S_{i}\in P_{j}}e^{M_{k}\cdot S_{i}}$$

Where *P_j_* is a specific promoter region sequence, *M_k_* is a specific PWM, and *S_i_* are all of the positions within the promoter region sequence. The score for any site with a given PWM is *M_k_*·*S_i_* and is related to the logarithm of the probability of the site being bound by the TF whose specificity is represented by the PWM (Chang *et al.* 2006; Granek and Clarke 2005; GuhaThakurta *et al.* 2004; Stormo 2000). The proportionality constant that relates this occupancy score to the true occupancy of the promoter region sequence is unknown but is not needed because we use a correlation coefficient to compare the occupancy score to the experimental determinations of binding locations.

A total of 57 binding assays, including array hybridizations (ChIP-chip, 39 samples) and sequencing (ChIP-seq, 18 samples), were obtained from the GEO database (Barrett *et al.* 2009). Individual samples are further processed where appropriate, such as comparing a specific ChIP-chip array to its control array or to different time points in a time series experiment. Thus, 51 experiments were used in the analysis, and a total of 794 motifs have a predicted occupancy that is significantly correlated (t-value > 6.02, *P* < 0.01 after correcting for multiple tests) with at least one of the 51 different processed samples (see supporting information, Table S1 for the complete list). An example is H01M10.2.1, which is significantly correlated to NFI-1 ChIP samples (t-value ~15.6, *P* < 1 × 10^−6^ after correction) (Whittle *et al.* 2009). Using ChIP-Seq Whittle *et al.* 2009 identified 55 genes that passed a strict cutoff for binding. The motif they identified in 49 of the 55 bound regions is nearly identical to our motif H01M10.2.1 and also to a previously reported motif for vertebrate NFIs (Figure 1). Of those 55 genes, 36 were included in the promoter region sequence sets analyzed by PhyloNet, and 32 of them had NFI-1 binding sites identified by Whittle *et al.* 2009 within the 2-kb upstream regions of our study. The PhyloNet PWM H01M10.2.1 contains 22 exemplar sites from that set of 32 reported NFI-1 sites (*P* < 10^−14^).

We also compared peaks from ChIP-seq experiments for 23 TFs as part of the modENCODE project (Gerstein *et al.* 2011). Table S1 lists the experimental sets (28 in total, including 6 for the TF Pha4 at different stages or different conditions). The type of DNA-binding domain is listed for each TF, and the consensus binding site if known. Most of these TFs do not have known binding sites, but for some the specificity can be inferred from orthologous TF in Drosophila (Zhu *et al.* 2011). The gene listed associated with the ChIP-seq peaks for each TF were compared to the exemplar gene set for each PhyloNet motif and significance of the overlap determined by a Fisher exact test. The top five motifs for each ChIP-seq dataset are listed along with their consensus sequences (Table S1). For 40% of the TFs with known and inferred binding motifs, there is a good match to at least one of the top PhyloNet motifs. Most of the TFs with known motifs but without a matching PhyloNet motif contain homeobox domains that have AT-rich, short, and degenerate motifs that may contribute to their inefficient discovery. For the majority of the TFs, without known binding motifs, the PhyloNet matrices represent potential motifs. However, most TFs, and especially those with low specificity, regulate genes in combination with other TFs. Because the associations shown in Table S1 are between gene lists, it may be that the motifs found for a particular TF ChIP-seq dataset actually correspond to different TFs that interact to coordinately control expression.

#### Expression analysis:

The same predicted occupancy scores for each PWM and each promoter region sequence can be compared with expression data to determine whether motif occurrences are significantly correlated with expression patterns. A total 1197 expression samples were obtained from the GEO database and further processed where appropriate, as in the location analysis. A total of 797 motifs are significantly correlated (*P* < 0.01 after correction for multiple tests) with at least one of 850 different processed samples (see Table S1 for the complete list).

In the aforementioned location analyses, we uncovered associations between specific motifs and the specific proteins that were immunoprecipitated. This lets us infer that the motif represents the binding specificity of the protein, or perhaps another protein that is tightly coupled to the one that is precipitated. In the expression analysis, we identify motifs that are associated with genes whose expression changes under different conditions, genetic backgrounds or at different times or different tissues during development. We can hypothesize that the motifs represents the binding sites for some proteins responsible for these changes in expression, but the identity of the proteins is usually unknown. However, in some cases we find the same motif identified in the location analysis and the expression analysis, which suggests that the specific protein acts through the identified motif to control the expression of the regulated genes. We find 424 such motifs that are significant in both datasets.

Although our collection of expression microarrays do not include any records in which NFI-1 mutants were probed for genome-wide expression, previous work suggests that NFI-1 is critical for wild-type adult lifespan (Lazakovitch *et al.* 2005). We observe significant correlations between occupancy scores for H01M10.2.1 on nearby genes and their expression changes in age-related micorarray records (*P* < 0.01). In addition, another discovered motif C18D1.3.5 is similar to the core portion of previously discovered motif Motif Enriched on X (MEX), which Jans *et al.* 2009 show to be a component of the dosage compensation complex. Interestingly, our location analysis results show that C18D1.3.5 is correlated with the Chip-Chip results for the dosage compensation complex subunits (DPY27: *P* < 10^−16^; SDC-2: *P* < 10^−16^; SDC-3: *P* < 10^−14^; MIX-1: *P* < 10^−3^; HTZ-1: *P* < 10^−16^). In addition, our expression analyses show that C18D1.3.5 matrix is also correlated with XO *vs.* XX-WT expression studies (*P* < 10^−16^).

Coregulated genes often have similar expression profiles under different conditions. We can thus evaluate the likelihood of a motif being biologically meaningful by the coherence of the expression profiles of all the target genes associated with the motif. We used the expression coherence score (Pilpel *et al.* 2001) to measure the overall similarity of the expression profiles of all the target genes of a given predicted motif in several different conditions. The NCBI GEO database contains nine datasets that studied *C. elegans* gene expression under different conditions or at different time points and therefore are suitable for expression coherence analysis. The nine data sets are PAL-1 network (GDS1319), Hypoxia response (GDS1379), TOM1/UNC-43 (GDS1786), Twist overexpression (GDS2463), lin-35−null mutant at various stages of development (GDS2751), Aging time course (GDS583), Heat stress time course (GDS584) Germline development (GDS6), and *daf-2* mutant expression profiling (GDS770). Using a stringency cutoff of *P* < 0.05 after correction for multiple tests, we determined that 682 (13.75%) exemplar gene sets exhibit similar expression patterns in at least one experimental condition, suggesting a regulatory function of that associated PWM (see Table S1 for the complete list). H01M10.2.1 matrix-associated genes, described previously as associated with NFI-1 binding, have significant expression coherence in both hypoxia response experiment and heat stress time course experiment. Interestingly, NF1 in Drosophila has similar functions of regulating life span as that of *C. elegans* NFI-1, and flies overexpressing *NF1* had increased life spans, improved reproductive fitness, increased resistance to oxidative and heat stress in association with increased mitochondrial respiration, and a 60% reduction in ROS production (Tong *et al.* 2007).

C47A10.6.1 is similar to the heat shock element identified in the promoter sequences of the genes that were consistently up-regulated 1 and 4 hr after heat shock (GuhaThakurta *et al.* 2002). Genes associated with C47A10.6.1 have significant expression coherence in heat stress time course experiment but not in any other experiment. F01G4.4.5 is similar to the heat shock associated site identified in the same study as heat shock element (GuhaThakurta *et al.* 2002). Similarly, genes associated with F01G4.4.5 have significant expression coherence in the heat stress time course experiment but not in any other experiment.

#### Tissue-specific expression patterns:

A total of 1882 *C. elegans* transcripts (∼10% of the genome) have classified expression patterns in 88 different spatial-temporal patterns between the larval and adult stages (Hunt-Newbury *et al.* 2007). Ignoring the developmental stage, we combined expression of the genes into 49 distinct tissue or cell-types. We asked whether the exemplar genes for any specific motifs were enriched for specific tissues with a Fisher exact test. After correcting for number of motifs and tissues, we find 251 motifs with genes that are significantly enriched in 23 of the 49 tissue and cell types. For example, the genes associated with the F26A1.1.1 PWM are enriched for pharyngeal genes (*P* < 5 × 10^−20^). This PWM is very similar to the known motif for the TF Pha4, which is known to direct transcription of pharyngeal genes (Gaudet and Mango 2002). In accordance with previous reports that NFI-1 is expressed in muscles (mainly pharynx and head muscles), neurons and intestinal cells (Lazakovitch *et al.* 2005, 2008), our corresponding motif (H01M10.2.1) is also enriched for genes whose GFP-fused promoters are expressed in the pharynx (*P* < 3 × 10^−5^) and body wall muscle (*P* < 7 × 10^−3^).

#### GO enrichments:

GO enrichment has been widely used to assess whether gene sets defined by various clustering methods appear to be significantly related to one another functionally. We compared the exemplar gene sets for each of the PhyloNet PWMs with the GO annotation, at a stringent significance threshold (*P* < 0.05 after correction for multiple tests), to find that 3676 (74%) are significantly enriched for at least one biological function.

In *C. elegans*, NFI-1 is shown to be import in regulating motility (Lazakovitch *et al.* 2005). Consistent with this, genes associated with H01M10.2.1 PWM are enriched for GO term microtubule cytoskeleton organization and biogenesis (GO:0000226, *P* < 10^−6^), microtubule organizing center (GO:0005815, *P* < 10^−5^), and microtubule-based process (GO:0007017, *P* < 10^−5^). Vertebrate NF1 is involved in chromatin/chromosome remodeling (Hebbar and Archer 2003) and *in vivo* target of *C. elegans* NFI-1 includes many genes involved in this process (Whittle *et al.* 2009). Consistent with this, genes associated with H01M10.2.1 PWM are enriched for GO term centrosome (GO:0005813, *P* < 10^−6^) and spindle organization and biogenesis (GO:0007051, *P* < 10^−8^). In addition, *in vivo* NFI-1 targets also includes phosphatase, vaculolar protein sorting factors, and protein translocation related proteins, and H01M10.2.1 PWM is enriched for GO terms phosphoserine phosphatase activity (GO:0004647, *P* < 10^−7^), vacuolar membrane (GO:0005774, *P* < 10^−7^), vesicle membrane (GO:0012506, *P* < 10^−5^), and protein transport (GO:0015031, *P* < 10^−5^). Taken together, the consistent evidence from multiple independent sources, that is, the similarity of H01M10.2.1 matrix to the *C. elegans* NFI-1 binding motif and the vertebrate NF-1 binding motif, significant enrichment in tissue-GFP analysis, location analysis, and expression analysis, as well as significant GO enrichment and NFI-1 targets enrichment, strongly suggests that our PhyloNet-discovered matrix H01M10.2.1 represents the DNA-binding specificity for NFI-1 TF.

If we combine all of the aforementioned biological assays described, we find that a large fraction (4066 of the 4959; 82%) of the predicted motifs have at least one type of evidence to support its regulatory function. Currently, most of the *C. elegans* TFs are uncharacterized, which limits our ability to make direct connections with the PWMs we discover with PhyloNet. However, the fact that all of the motifs are conserved across species as well as highly similar in the regulatory regions of multiple genes, and the fact that a large fraction of them are supported by one or more types of experimental or comparative evidence, leads us to believe that they represent regulatory sites for one, or more, TFs and control the expression of *C. elegans* genes.

### Using the motifs to predict CRMs

A CRM is a segment of DNA that generally contains multiple TF binding sites that function together to regulate the particular expression patterns of the associated gene. Many studies have shown that in higher organisms, CRMs are a common strategy in regulating gene expression. If our predicted motifs are functional, we would expect the exemplar sites composing those motifs to overlap significantly with experimentally defined regulatory modules. From the literature, we collected 41 promoter region sequences from the 9536 genes included in our PhyloNet analysis that have been experimentally tested for the location of regulatory regions. The experiments involve inserting segments of promoter region sequences into vectors to create transgenic worms, and then it is determined whether that region drives expression of a reporter gene, typically GFP. Often the promoter sequence segments that are tested are large and don’t provide finer resolution about the critical region, but in other cases the tested segments were small or deletions were introduced to identify critical regions.

Using the 2-kb upstream sequence of the 41 genes gives us 82 kb of potential regulatory sequence for our comparison. There are a total of 61 CRMs that have been experimentally determined in those regions, covering a total of 26,594 bp, or 32.4% of the total sequence. This undoubtedly contains regions that are not essential for activity, but that is the limit of the resolution from the currently available experiments. The 41 promoter region sequences contain a set of 12,107 exemplar sites and cover 12,473 bp, or 15.2% of the total sequence. If those two sets of sequences were unrelated, we would expect them to overlap by approximately 5% of the total promoter region, but in fact the overlap is much higher. Of the 61 experimentally confirmed CRMs, 53 (86.9%) of them have overlapping exemplar sites, indicating that using exemplar sites to predict CRMs would have high sensitivity. A total of 6428 (53.1%) of the exemplar sites for these genes are within experimental CRM regions, which is the minimum positive predictive value (PPV) of the exemplar sites. It could be much higher because not all regions of the promoter sequences were tested and there could be additional CRMs in the promoters that are also functional. These results together indicate that exemplar sites from the PhyloNet analysis can be used to accurately predict the likely regulatory regions for many *C. elegans* genes.

**Figure 2:**
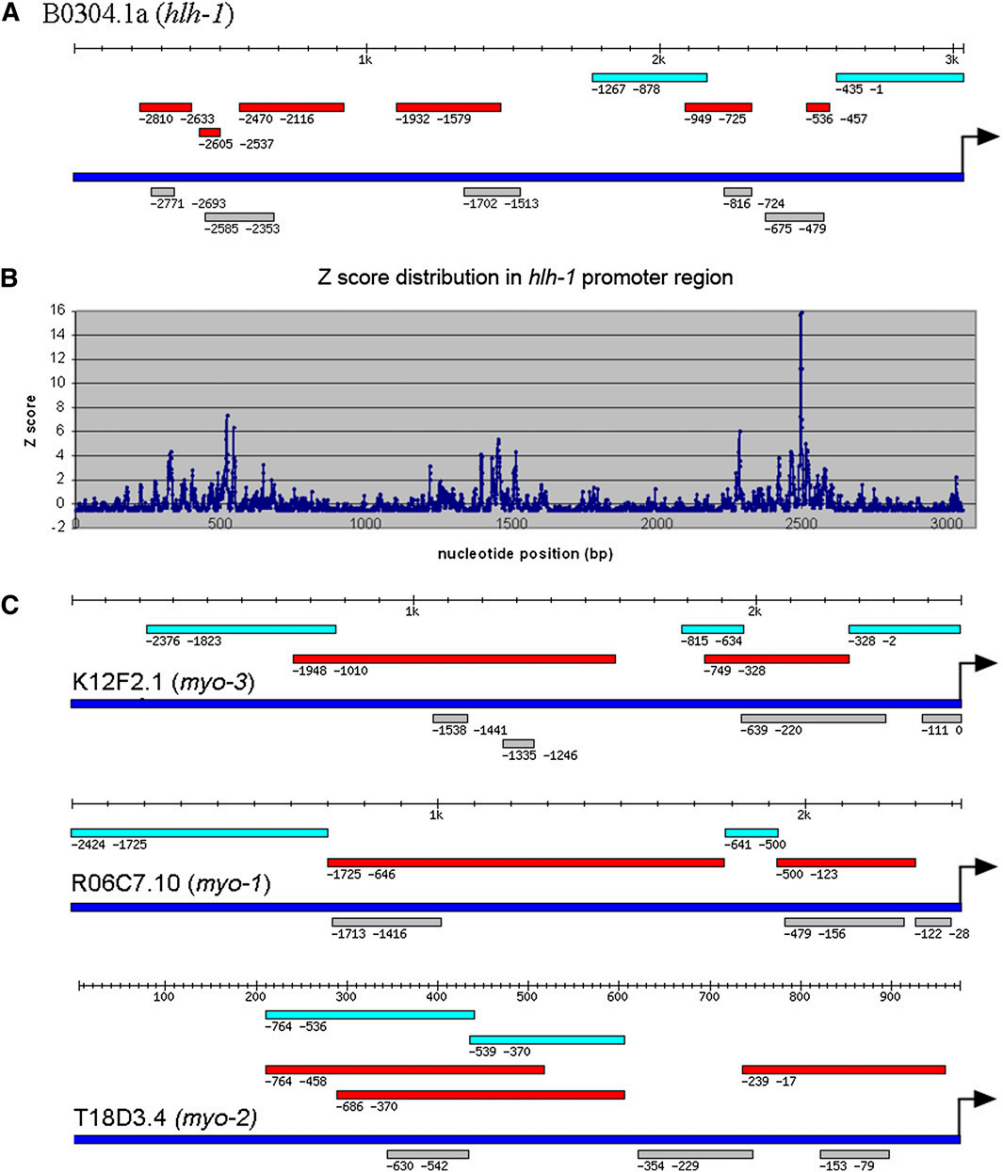
Comparison between predicted CRM with experimentally defined CRM in four best studied promoters. (A) Comparison between predicted CRM with experimentally defined CRM in *hlh-1*(Krause *et al.* 1994). (Turquoise bar: experimentally tested DNA fragment without regulatory function; red bar: experimentally tested DNA fragment with regulatory function; deep blue bar: promoter sequence; gray bar: predicted CRM. Arrow: translational start codon. Position coordinates shown are relative to translational start codon.) (B) Distribution of Z score of number of motif sites across the *hlh-1* promoter region. (C) Comparison between predicted CRM with experimentally defined CRM in *myo-3*, *myo-1*, and *myo-2* (Okkema *et al.* 1993). (Labeling the same as in A.)

We can also predict CRMs based on predicted binding sites using the PWMs. Although this will increase the false positive rate, it allows predictions across the whole genome, not just the ~50% of genes used in the PhyloNet analysis and not limited to the 2-kb upstream region. We find that the predicted binding sites based on the PWMs are highly clustered along the promoter sequences (Figure 2B), which is consistent with previous experimental observations and the general model that DNA sequences with clustered TF binding sites are usually regulatory sequences that direct specific spatial and temporal gene expression (Arnone and Davidson 1997; Blanchette *et al.* 2006; Sinha *et al.* 2006; Wasserman and Sandelin 2004). To examine whether DNA regions with significantly enriched motif binding sites correspond to regulatory sequences, we focused on regions that have binding sites significantly more than average (Z score ≥ 3.09, *P* ≤ 10^−3^). For example, *hlh-1* (B0304.1) upstream sequence is one of the best studied promoter regions. A total of six regulatory sequences are identified by detailed deletion and enhancer assays (Krause *et al.* 1994). The regions with significantly enriched motifs correspond very well to the experimentally delineated regulatory sequences (Figure 2, A and B). Based on this observation, we developed an algorithm, *C. elegans* Regulatory Module Detector (CERMOD), to predict regulatory modules using the 4959 PhyloNet PWMs. For *hlh-1*, CERMOD predicted five modules in the full 3053 bp upstream sequence that overlap all six known regulatory sequences (Figure 2A).

To evaluate the predictive power of CERMOD, we performed a thorough literature search to identify any *C. elegans* genes whose promoter regions have been analyzed to locate any regulatory sequences. We identified 79 genes that are expressed in a broad range of tissues at various developmental times (Table S1). We analyzed upstream intergenic sequences, which range from 347 bp to 20,000 bp. There are 155 experimentally determined regulatory regions that are important for the corresponding gene expression in neurons, hypodermis, excretory cells, muscle precursor cells, adult muscle cells, vulva cells, sheath cells, and others. These regulatory regions are determined by deletion and/or enhancer assays. Wherever possible, we use regions that are determined by enhancer assay because it better defines the boundary of regulatory regions that are sufficient in regulation. Application of CERMOD on this set of data identified 129 of the 155 (83.2%) experimentally defined modules. Figure 2, A and C shows the comparison between predicted modules with experimentally defined modules in the upstream sequences of four well-studied genes (Krause *et al.* 1994; Okkema *et al.* 1993). Because some of the predicted modules are located within DNA sequences that have not been tested, we cannot calculate the PPV but it is at least 23.5% (219/934). The real PPV is surely higher because in some studies reporter gene expression is not reported for additional tissues (Wenick and Hobert 2004). Table S1 shows the comparison of predicted and experimentally characterized modules in the entire set of genes with experimental evidence.

We performed simulations to estimate the statistical significance of obtaining the same sensitivity and PPV given the promoter sequences and the known regulatory modules. We simulate the distribution of predicted modules in the promoter region sequences by randomly picking a start position for each module. The length and number of modules in each gene is kept the same as the predicted modules in this gene. The simulation is repeated 10,000 times, and the sensitivity and PPV are calculated for each one. The average sensitivity is 62.4% with standard deviation of 3.3%. The average PPV is 19.6% with standard deviation of 1.1%. Therefore, the p-values of getting 83.2% sensitivity and 23.5% PPV are both much less than 0.001.

Because many experimental modules have not been further analyzed to delineate the boundary, the functional module can be very long (experimental modules referenced in this manuscript range from 44 to 5287 bp). This resulted in high sensitivity in simulated data. To reduce the effect of those long experimental modules, we used only modules that are within the size range of predicted modules (27-580 bp) and recalculated the sensitivity and PPV. The sensitivity changed very little (72 of 92 are correctly predicted, 78.3%) but the sensitivity on the simulated data are greatly reduced (48.3%), making our predictions even more significant.

#### CRM prediction in miRNA promoter region sequence and introns:

**Figure 3:**
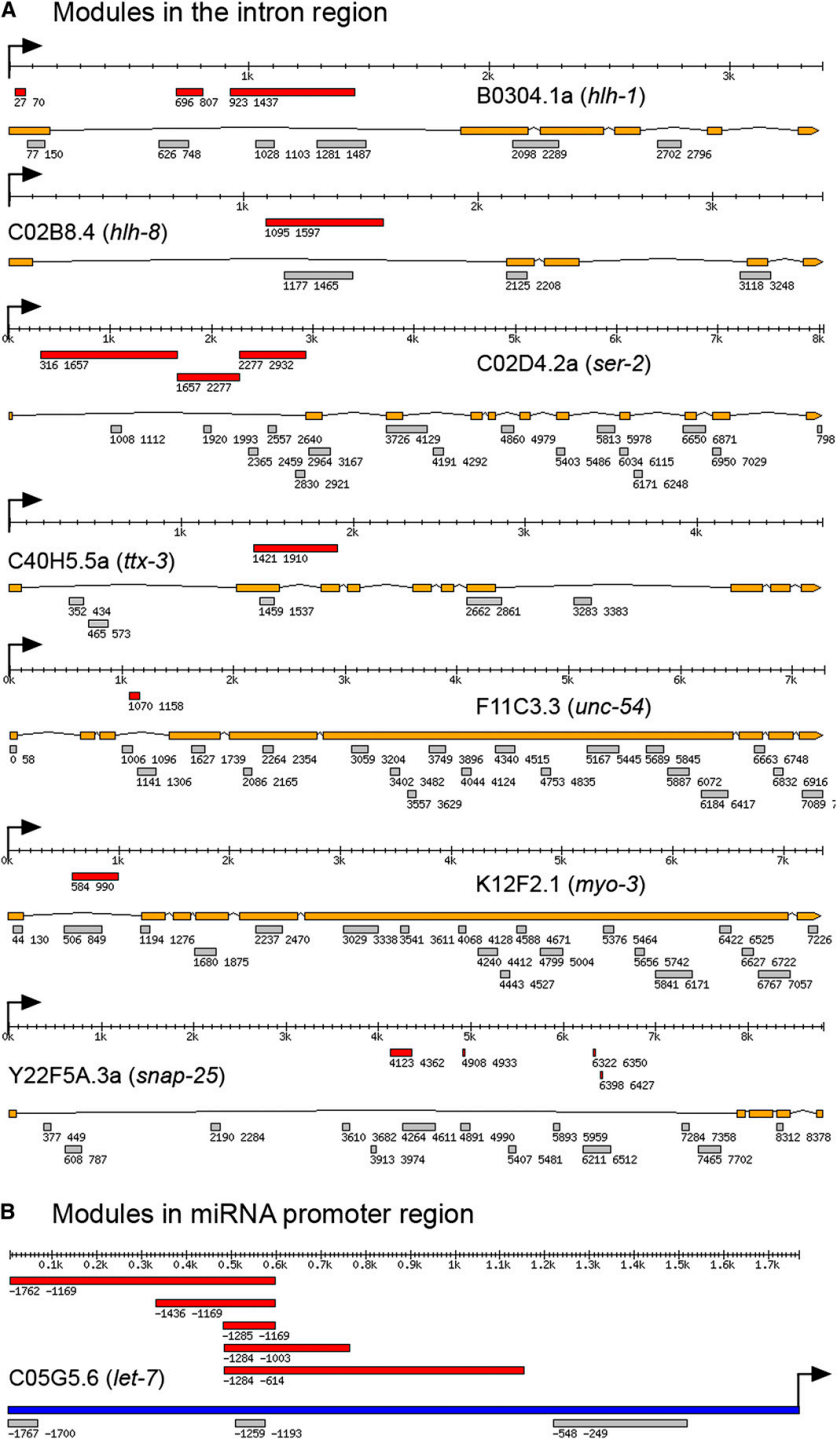
Comparison between predicted CRM with experimentally defined CRM in intron regions and in miRNA *let-7* promoter region. (A) Comparison in intron regions. (B) Comparison in miRNA *let-7* promoter. Red bar: experimentally tested DNA fragment with regulatory function; orange bar: gene exons; gray bar: predicted CRM; deep blue bar: promoter sequence. Arrow: translational start codon. Position coordinates shown are relative to translational start codon.

Experiments have shown that some introns contain transcriptional regulatory sequences (Hwang and Lee 2003; Krause *et al.* 1994; Okkema *et al.* 1993). To test whether CERMOD can predict CRMs in intronic regions, we identified seven genes in which intronic regulatory sequence have been mapped in detail. There are 14 experimentally defined modules in the introns from these seven genes, 10 of which are correctly predicted (71.4%; Figure 3A). We performed simulations as described previously and the simulated data has an average sensitivity of 46.0%, making our predictions highly significant (*P* < 0.005).

microRNAs (miRNAs) are ~22 nt RNAs that bind to partially imperfectly matched sites on target mRNAs to regulate transcript expression. They are now known to influence a broad range of biological processes. However little is known about how miRNA transcription is regulated. Currently there is only one miRNA, *let-7*, whose promoter region sequences has been dissected to identify regulatory sequences. The *let-7* family of microRNAs, first discovered in *C. elegans*, is functionally conserved from worms to humans. A growing body of evidence suggests that the human let-7 expression is misregulated in many human cancers, and restoration of *let-7* expression may be a useful therapeutic option in cancers (Boyerinas *et al.* 2010). Expression of *let-7* RNA is temporally regulated with robust expression in the fourth larval and adult states. Several DNA fragments were tested, and the DNA fragment located at [−1169, −1285] upstream of the mature RNA was identified as the minimal DNA fragment that is necessary and sufficient for this temporal regulation. We predicted three modules in the ~1.8-kb upstream sequence (Figure 3B). The predicted module [-1193, −1259] overlaps with the experimentally defined minimal regulatory module [−1169, −1285]. *let-7* is also expressed in the anchor cell at L3 and in the distal tip cells at the adult stage (Esquela-Kerscher *et al.* 2005). It would be interesting to see whether the other two predicted modules drive *let-7* expression in those cells.

#### Experimental test of CRM prediction:

**Figure 4:**
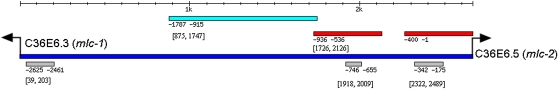
Experimental test of predicted CRM in *mlc-1/mlc-2* intergenic region. Turquoise bar: DNA fragment tested that did not show regulatory function; red bar: DNA fragments that showed regulatory function; deep blue bar: *mlc-1/mlc-2* intergenic region DNA sequence; gray bar: predicted CRM. Arrow: translational start codon. Positive position coordinates shown are relative to *mlc-1* translational start codon. Negative position coordinates shown are relative to *mlc-2* translational start codon.

*mlc-1* and *mlc-2* are the two muscle regulatory myosin light chain genes in *C. elegans*. They are divergently located and share a 2.6-kb intergenic region. It was shown that they are both expressed in the body-wall muscles, pharyngeal muscles, and vulval muscles. However, the intergenic region has not been analyzed in detail to identify all the regulatory sequences that drive their expression. Previous study has shown that the first 400 bp of *mlc-2* upstream sequence is enough to drive its expression in the body wall muscle cells (GuhaThakurta *et al.* 2004). To gain better information about transcriptional regulation of *mlc-1* and *mlc-2*, we applied our module prediction method on the intergenic region of *mlc-1/mlc-2* and experimentally tested our prediction. Within this 2662 bp DNA fragment our method predicted 3 CRMs (Figure 4). Positive position coordinates shown are relative to *mlc-1* translational start codon. Negative position coordinates shown are relative to *mlc-2* translational start codon. DNA sequence between 39 and 203 bp ([39, 203]) is just upstream of *mlc-1*; DNA fragment [1918, 2009] is located at −655 to −746 bp upstream of *mlc-2* translational start codon; DNA fragment [2322, 2489] is close to *mlc-2* translational start codon ATG and corresponds to the first 400 bp upstream that we had previously shown to drive expression in the body wall muscle (GuhaThakurta *et al.* 2004). We tested DNA sequence from 1726 to 2126 ([1726, 2126]), which includes one of the predicted CRMs, and DNA sequence from 875 to 1747 ([875, 1747]), which does not include any predicted CRM, for enhancer activity by cloning them into a *pes-10* minimal promoter (Fire *et al.* 1990). Only the DNA fragment that covers the predicted module showed enhancer activity and the expression was limited to the pharyngeal muscle. Therefore, these two experimental results are consistent with the use of PhyloNet PWMs for predicting regulatory regions of *C. elegans* promoters. This result has another interesting aspect. The *mlc-2* gene is known to be expressed in both body wall and pharyngeal muscle, and we have separated those two tissue-specific expression patterns into two separate CRMs. The closest enhancer upstream of the ATG drives expression only in body wall muscle, and the farther enhancer, located over 500-bp upstream, drives expression in the pharyngeal muscle.

### Facilitating access to the sites, motifs, and module predictions

All data and results discussed here, including the putative regulatory motifs, supporting evidence for each motif, list of motifs that are significant in each analysis, experimental modules and references, pictures of experimental modules and predicted modules in promoter regions, as well as in intron regions and microRNA promoter region sequences are available via the web interface at http://stormo.wustl.edu/gzhao/CE_PhyloNet/. Each motif can be accessed by name and the link provides the exemplar sites and the gene list for that motif as well as other related information. Links are also included for all of the genes containing exemplar sites where all of the motifs they are associated with can be found.

**Figure 5:**
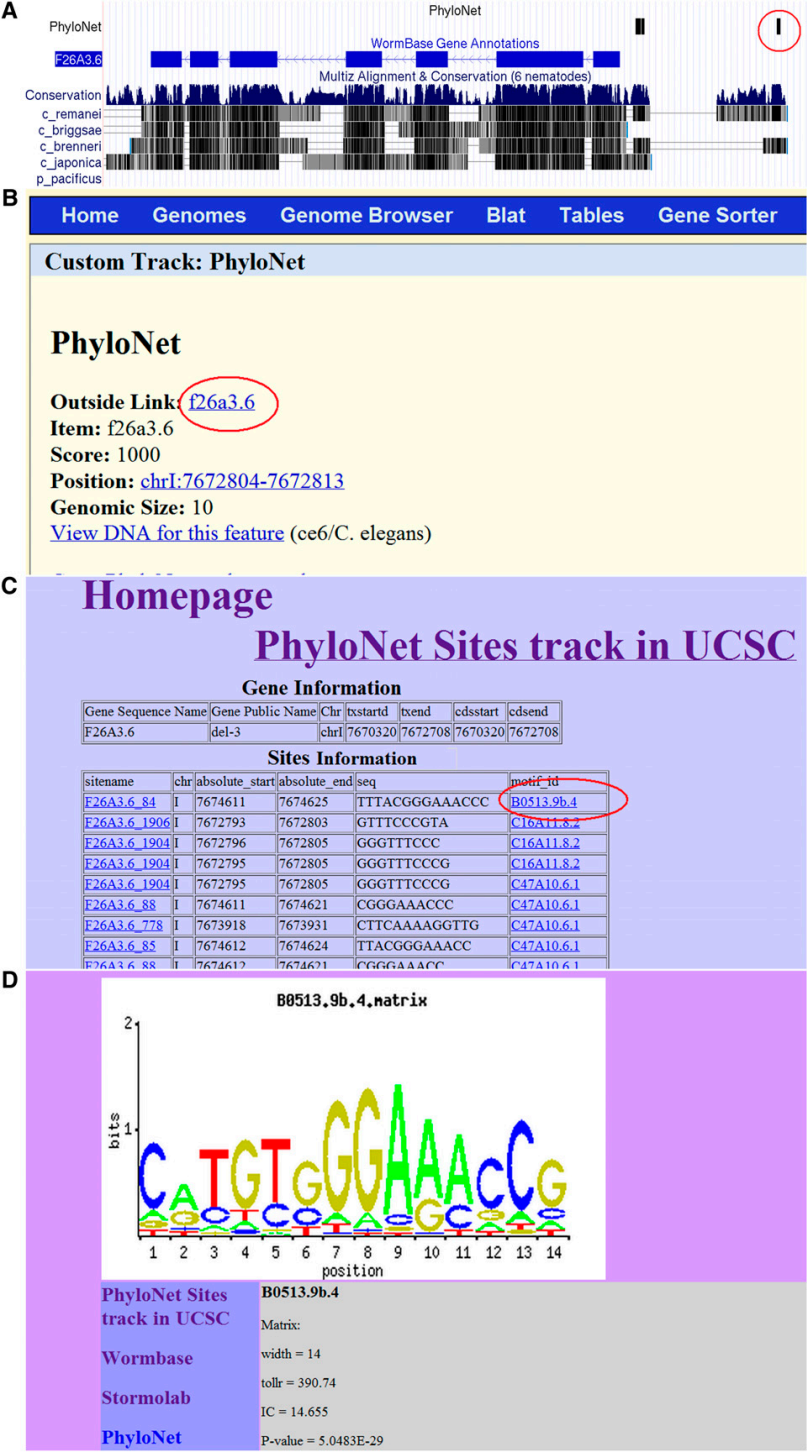
Example use of the UCSC genome browser. (A) Screen shot of genomic region containing exemplar sites; clicking on the red circled exemplar site in front of the gene F26A3.6 takes you to the additional information page for this gene, shown in (B). Clicking on the outside link (highlighted in red) takes you to a table with all the motifs in this promoter region, shown in (C). Clicking on the specific motif highlighted in red opens a new page displaying the additional information for this motif, shown in (D).

We have created files for both the exemplar sites and CERMOD predicted modules across the whole genome in BED formats that can be uploaded as custom tracks and viewed in the UCSC genome browser. These track records can be downloaded from http://stormo.wustl.edu/gzhao/CE_PhyloNet/. In the genome browser page, when a PhyloNet site or CERMOD module is clicked on, it opens up an external site with information about the motif or module. For a motif, this information includes a logo of the motif, the matrix and all exemplar sites genome-wide. Figure 5 highlights the capabilities of this interface.

### Conclusions

We performed a genome-wide search for conserved regulatory elements in *C. elegans*, *C. remanei*, and *C. briggsae* and identified a total of 4959 regulatory elements. Our study identified regulatory elements with diverse biologically functions that include at least core promoter elements, TF binding sites, and functional RNA sites. Multiple independent evidence provide strong support for their biologically significance. The distribution of these regulatory motifs along promoter region sequences is highly clustered, which allowed us to accurately detect DNA regulatory sequences that drive spatial/temporal-specific gene expression. Our work greatly expanded our knowledge of regulatory sites in *C. elegans* and is a valuable step toward building a genome-wide regulatory network of *C. elegans*. CERMOD predicts modules from the distribution of the predicted motif occurrences along the promoter region sequences and identifies statistically significantly clustered motif sites. It does not require a training set and it is not necessary to know in which tissue a gene is expressed. It has high sensitivity and specificity on experimentally verified CRMs, and we expect it to have similar sensitivity and PPV on any given *C. elegans* sequence. The accessibility of all of our results, the exemplar sites, the predicted motifs, and the predicted CRMs, through the UCSC genome browser should make them a valuable resource for the research community.
